# Systems biology during 20 years of PLoS Computational Biology

**DOI:** 10.1371/journal.pcbi.1014465

**Published:** 2026-07-10

**Authors:** Mark Alber, Marc R. Birtwistle, Stacey D. Finley, Pedro Mendes

**Affiliations:** 1 Department of Mathematics and Interdisciplinary Center for Data-driven Modeling in Biology, University of California, Riverside, Riverside, California, United States of America; 2 Department of Chemical and Biomolecular Engineering, Clemson University, Clemson, South Carolina, United States of America; 3 Departments of Biomedical Engineering and Quantitative & Computational Biology, University of Southern California, Los Angeles, California, United States of America; 4 Center for Cell Analysis and Modeling, University of Connecticut School of Medicine, Farmington, Connecticut, United States of America; Johns Hopkins University, UNITED STATES OF AMERICA

As *PLoS Computational Biology* turns 20 years old, we reflect on the growth and evolution of Systems Biology both in general and as it relates to papers published here, with particular emphasis on the last 5–7 years. While the Systems Biology section at *PLoS Computational Biology* did not officially start until 2017, the journal and the field arguably grew together, and many influential Systems Biology papers were published here along the way. This perspective, of course, does not have the capacity to cite all such papers, but we hope that the few selected support the narrative and make the case that Systems Biology is now a mature field that continues to exert influence on the research being done across many modern biological and medical disciplines.

**Fig 1:**
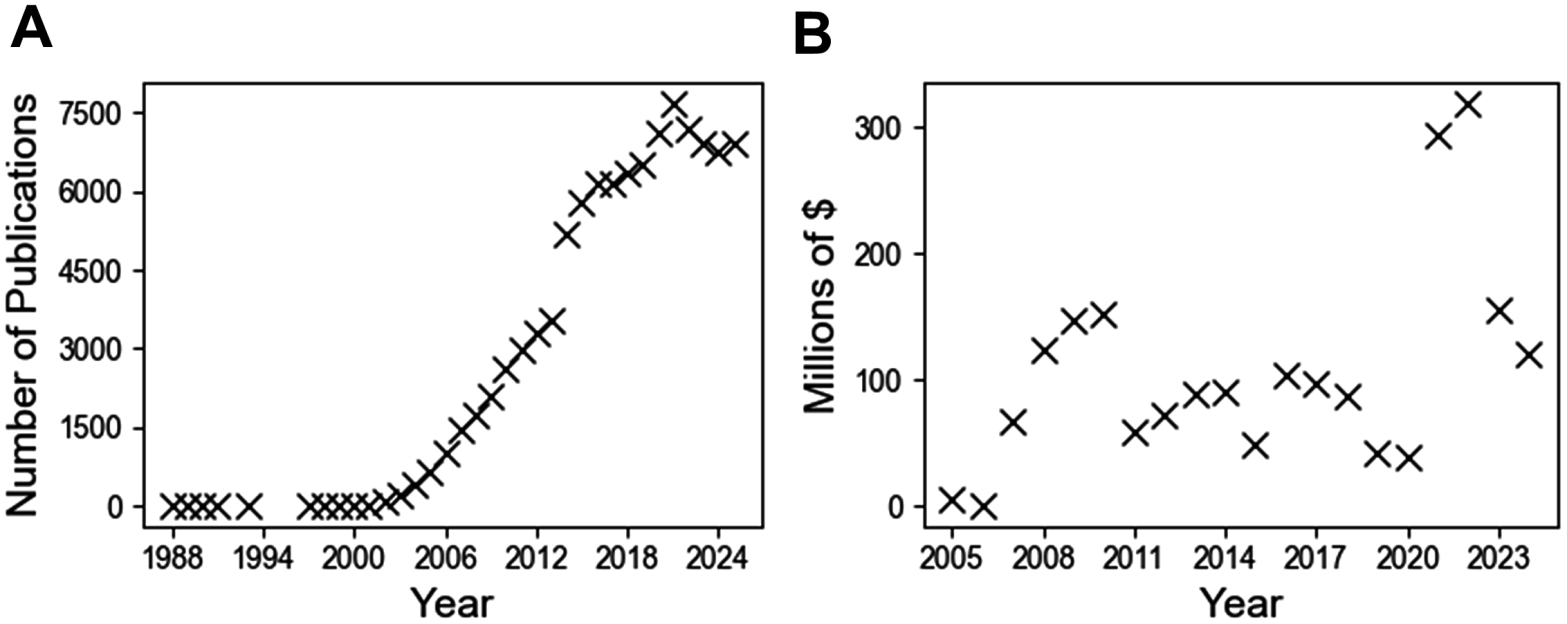
Systems biology trends. **A.** PubMed was queried for all manuscripts containing the phrase “systems biology”. Year 2025 data were removed. **B.** United States government funding data was retrieved from USASpending.gov, querying for awards containing “systems biology”. The column “total_funding_amount” was summed over years. Year 2025 data were removed.

To start, it is instructive to reflect on the history and definitions of Systems Biology, which started becoming recognized as a field in roughly the year 2000, when the first International Conference on Systems Biology (ICSB) was held, and a new research institute, the Institute for Systems Biology, was established in Seattle [1]. In the early years of this century, articles and worldwide government funding awards started to appear containing the phrase “systems biology”, which grew rapidly shortly thereafter (Fig 1—US funding shown for illustration). This includes the German Virtual Liver Network, UK Centres for Systems Biology, Japanese Erato-sponsored projects, and others. Similarly, departments, training, and education programs in Systems Biology began to emerge around the globe, such as the Harvard Department of Systems Biology, the joint Pittsburgh/Carnegie Mellon Computational and Systems Biology program, and the Infrastructure for Systems Biology in Europe. These programs originally stemmed from a combination of existing disciplines such as biology, medicine, engineering, physics, chemistry, computer science, and applied mathematics. Now, the programs are composed of and producing true interdisciplinary researchers.

Descriptions of the field of Systems Biology often emphasize development and application of predictive, quantitative, data-driven and multi-scale models, integration of multi-omics datasets, and interdisciplinary science and medicine, with a translational focus. The birth of the field coincidentally (or not so coincidentally) aligned with the completion of the human genome project, which gave us a so-called “parts list” and promised to solve many outstanding questions related to human biology. However, the question of how these parts interact and give rise to function was predominantly unresolved, which falls squarely into the purview of Systems Biology. We think many would still agree with such a characterization of Systems Biology today. Moreover, the field includes the study of the spatiotemporal dynamics of biological processes across length, time, and biological scales, with an emphasis on mechanistic interpretation. That being said, the lines are blurry between Systems Biology and many of the other journal sections, underscoring the overall interdisciplinary aspect of computational biology. We would, in addition, like to draw some distinction between Systems Biology and bioinformatics. Although there are many similarities, just as with other computational biology sub-disciplines, we would define bioinformatics as often dealing exclusively with sequence-based datasets and/or statistical analysis, and not necessarily with quantitative, mechanistic, spatiotemporal, and/or multi-scale descriptions thereof.

One area that *PLoS Computational Biology* has always been deeply vested in is modeling standards, reproducibility, and sharing, many times communicated through the popular 10 Simple Rules [2–5]. Giving a voice to highlighting serious potential flaws in status quo analyses, such as those involved with current single-cell genomics, is an important focus [6]. Systems Biology in many ways co-evolved with the development of standards such as SBML [7] for building and BioModels [8] for sharing reproducible computational models, efforts which further grew into proposed community best practices primarily adopted by and to some extent originally reported by this journal [9–11]. Some of this growth in the scale of models and available data has necessitated the adoption of best practices from computer science, where Systems Biology models are as much sets of equations as they are software packages. *PLoS Computational Biology*, therefore, has also been a home to papers describing new software in this field [12] and to research focused on model sharing, standards, reproducibility, and rigorous analysis [13].

Related to the above is parametric uncertainty and its effect on prediction uncertainty. One particular paper proposed the idea that parametric uncertainty may not be the best metric to focus on, because many individual parameters in models may have large ranges in which they can vary, yet the biological system maintains robust dynamic outputs [14]. This topic remains quite vigorously debated to this day. Indeed, methods to guarantee parametric identifiability, and to quantify Systems Biology model uncertainty remain actively researched and, as yet, difficult to achieve. Paradoxically, as artificial intelligence/machine learning (AI/ML) becomes an inescapable part of Systems Biology and nearly all areas of research, we observe that the number of free parameters in AI/ML models can often be on the order of 10^9^. Thus, reconciliation of the implications of parametric uncertainty in mechanistic computational models versus statistical AI/ML models remains to be seen. Parametric and prediction uncertainty also have implications in a range of contexts, for example, when considering translational or clinical applications [15,16] and design tools for synthetic biology [17–20].

Much Systems Biology research evolved with the available data, such as the development and subsequent improvement of the microarray for transcriptome profiling [21]. Quite early, the field began to focus on single-cells and their variability, as opposed to population averages provided by experimental methods profiling bulk behavior. Fundamentally distinct inferences about system structures were found by looking at single-cell dynamics. For example, cell-to-cell variability in protein expression (so-called intrinsic noise in gene expression) was and is still thought of as a key driver of why clonal cells can have different outcomes when presented with the same perturbations. This phenomenon has been studied broadly [22,23] and in specific contexts such as apoptosis [24,25]. Differences in epigenetic states, often called cell states, became a focus for explaining such behavior as well, and their identification from data remains an important research area [26]. Scaling “bottom-up” descriptions to larger models incorporating more biology and data became possible with improvements to deterministic solvers [27] and hybrid approximations [28], and to agent-based modeling software that enables examining such phenomena in spatial context [29]. Pioneering large-scale, bottom-up approaches were focused on genome-scale metabolic models that describe fluxes of metabolic reactions based on fundamental gene- and metabolite-level knowledge combined with metabolomics (and other -omics) data [30,31]. High resolution microscopy imaging provides another rich data source to construct Systems Biology models applied in cell and developmental biology. For example, imaging data has been used to study cell morphological changes in multi-scale mechano-signaling models [32,33]. While the integrated multi-pathway and stochastic nature of biological systems across many scales is clearly important, adding such features to models makes identifiability and other issues discussed above even more challenging [34,35]. We expect this to remain an important and active area of Systems Biology research that *PLoS Computational Biology* will welcome.

Along the lines of the field evolving with available data, so too did the scale at which many models were developed, including so-called “top-down” models, distinct from bottom-up approaches described mainly above, in that they are predominantly statistically-derived but yield mechanistic predictions that describe topological connections amongst proteins and/or other types of biochemical molecules [36]. Consistent with this idea, for *PLoS Computational Biology* article titles from 2018 to 2024 in the Systems Biology section, the most obvious temporal change is that the word “data” has increased in frequency recently and “system” seems to have declined. Throughout all years, the words “cell” and “model” and “network” dominate (with “dynamic” and “cancer” also substantial). Top-down, data-driven approaches are fairly obvious candidates for integration with modern AI/ML approaches, which have been emphasized in *PLoS Computational Biology* for independent analysis and for integrating and/or improving mechanistic models [37]. The fact that the increased frequency of the word “data” in *PLoS Computational Biology* article titles correlates with the large rise in machine learning approaches across the scientific literature is perhaps not particularly surprising. In fact, one of the most cited recent articles in the Systems Biology section is based on coupling multi-scale mechanistic and AI/ML modeling [37]. Indeed, AI/ML approaches are used in many applications within Systems Biology. For example, genome-scale metabolic models can be improved by leveraging machine learning [38]. ML approaches are also used for developing personalized models and digital twins in biology and healthcare [39–42]. The rapidly growing arsenal of AI/ML tools is expected to continue to have an impact on Systems Biology research and be of interest to *PLoS Computational Biology*, particularly if combined with mechanistic modeling. This combination has the advantage of combining the inference power of AI/ML and the explainability and extrapolation power of mechanistic models.

In conclusion, *PLoS Computational Biology* grew up and came of age in parallel with the growth of Systems Biology. The field of Systems Biology is now quite a mature and established science that is intertwined with most, if not all, biological and even clinical sciences. We are enthusiastically awaiting the submission of the next wave of papers that will push the cutting edge of the field. In particular, we expect advances in how AI/ML can be integrated with mechanistic approaches to push the boundaries of quantitative, dynamic, and multi-scale modeling. However, we also expect that such advances would be in lockstep with robust, rigorous, reproducible, and sharable approaches, as discussed above. Thus, we expect boundary-pushing contributions in those regards as well, independent of AI/ML. Most importantly, we view these technical advances as a means to learn new biology and solve clinical problems that otherwise would be difficult, if not impossible, without Systems Biology. We are excited to see how *PLoS Computational Biology* contributes to the growth of the Systems Biology field in the coming years.
